# What sets the mutation rate of a cell type in an animal species?

**DOI:** 10.1371/journal.pbio.3003799

**Published:** 2026-06-08

**Authors:** Marc de Manuel, Molly Przeworski, Natanael Spisak, Anastasia Stolyarova

**Affiliations:** 1 Institute of Evolutionary Biology, CSIC-Universitat Pompeu Fabra, Barcelona, Spain; 2 Department of Biological Sciences, Columbia University, New York, New York, United States of America; 3 Department of Systems Biology, Columbia University, New York, New York, United States of America; 4 Institut Imagine, INSERM, Paris, France

## Abstract

Germline mutation rates per generation are strikingly similar across animals, despite vast differences in life histories. Analogously, in at least one somatic cell type, mutation burdens at the end of lifespans are comparable across mammals. These observations point to a key role for natural selection in shaping mutation rates. This Essay summarizes the patterns identified to date and outlines existing theories for how selection pressures might shape mutation rates in animal germline and soma. An understanding of what sets the mutation rate of a given cell type in a species requires better integration of genetics and development with population processes of selection and genetic drift.

## Abstract 2

Mutation rates per generation are strikingly similar across germlines of animals and across at least one somatic cell type, suggesting a key role for natural selection in shaping mutation rates. This Essay summarizes the patterns identified to date and outlines existing theories for how selection pressures might shape mutation rates in animal germline and soma.

## Introduction

Germline mutations arise from accidental changes to the genome in a cell lineage leading to egg or sperm and, when transmitted to an offspring, are present in every one of their cells. They are the substrate of adaptation; without them, there would be no evolution. Yet their net effect is deleterious [1,2]: while most de novo mutations are without fitness effects and a tiny fraction are advantageous to the organism, a larger number are instead harmful, introducing perturbations that lead to miscarriages, cause developmental disorders, and contribute to heritable diseases. In humans and other species with dedicated germlines, somatic mutations are restricted to a subset of tissues or cells; while most are inconsequential, some drive cancer and contribute to the aging process [3,4]. Given these deleterious effects, the existence of mutations presents somewhat of a paradox [5].

Mutations are not only inputs into disease and evolution, however, they are also outputs. Mutations in both germline and soma arise from the interplay between myriad sources of damage, DNA replication cycles, and repair and damage tolerance pathways, involving hundreds of genes [6]. Human mutation rates are heritable (controlling for parental ages; [7,8]) and the mutation spectrum evolves over time (e.g., in humans; [9,10]). Viewed through this lens, mutation rates in germline and soma are coupled quantitative traits, subject to genetic drift (Box 1) and natural selection. How these forces combine to set the mutation rate of a species has been of interest for close to 90 years [1,2,11,12] and is central to the study of aging as well as of evolution.

**Box 1. Glossary.**

**Genetic drift**

The random fluctuations in allele frequencies that occur over time in a finite population and lead to evolution in the absence of natural selection.

**Effective population size (*N*_e_)**

Often invoked in population genetics, the parameter *N*_e_ refers to the size of a model population that would experience the same magnitude of genetic drift as observed in the study population. Natural selection against a given deleterious allele is less effective in species with smaller effective population sizes (because the species experiences greater genetic drift).

**Generation time effect**

The long-standing observation in evolutionary biology that larger, longer-lived species have a lower germline mutation rate per unit of time than do smaller, shorter-lived species. This pattern was first inferred from phylogenetic data and has been confirmed by pedigree sequencing.

**Paternal bias**

Sometimes called male bias. The ratio of de novo germline mutations transmitted by fathers versus mothers. Across mammals and other classes of vertebrates, the paternal bias is greater than 1; in humans, it is 3–4.

**Modifier allele**

Sometimes referred to as mutator allele. In the context of mutation rate evolution, a modifier refers to an allele that alters the mutation rate at other sites in the genome (e.g., by changing the accuracy of a DNA repair mechanism).

**Driver mutation**

A mutation that confers a selective advantage to a cell (e.g., by increasing the rate of proliferation) and contributes to cancer initiation or progression.

**Gompertz law of mortality**

A demographic principle that refers to the exponential increase in mortality rates with advancing chronological age in adult populations.

**COSMIC mutational signatures**

Characteristic patterns of mutations, inferred from a large dataset of tumor genomes [13,14]. The idea is to model mutations in a given sample from a cancer or a normal tissue as a combination of relatively few signatures, each of which reflects a distinct mutational process. For single-base substitutions, a signature is defined by a distribution over the 96 possible mutation types, given by the substitution and its flanking bases. Signatures and their properties are listed in the catalog of somatic mutations in cancer (COSMIC) database; a subset has been assigned a known etiology.

To date, theories about the selective pressures acting on germline mutations have been motivated by a few ‘stylized facts’. First, comparisons across prokaryotes, unicellular, and multi-cellular organisms indicate that taxa with larger effective population sizes (Box 1) have lower mutation rates per base pair (bp) per generation [2]. Second, mammalian genomes of short-lived species accumulate more mutations per unit time than do those of longer-lived species [15–17]. Third, in humans and other amniotes, fathers transmit more de novo mutations than mothers, reflective of a higher mutation rate in spermatogenesis relative to oogenesis [18–21]. These broad strokes have stood the test of time, but can now also be filled in with numerous, direct estimates of germline mutation rates from pedigree studies, as well as mutation rates for diverse tissues and somatic cell types in humans as well as other species.

The recent data reveal some striking patterns, including the similarity in germline mutation rates per generation seen across animals and the constancy in mutation rates at the end of life span in intestinal crypts, as well as a seeming deviation from this trend in blood stem cells. In this Essay, we outline the picture that is emerging and discuss existing theories about the selection pressures that might give rise to patterns in germline and soma; throughout, our emphasis is on how the models might be refocused to consider differences among cell types.

## Mutation rates in mammals: some observations

**Fig 1:**
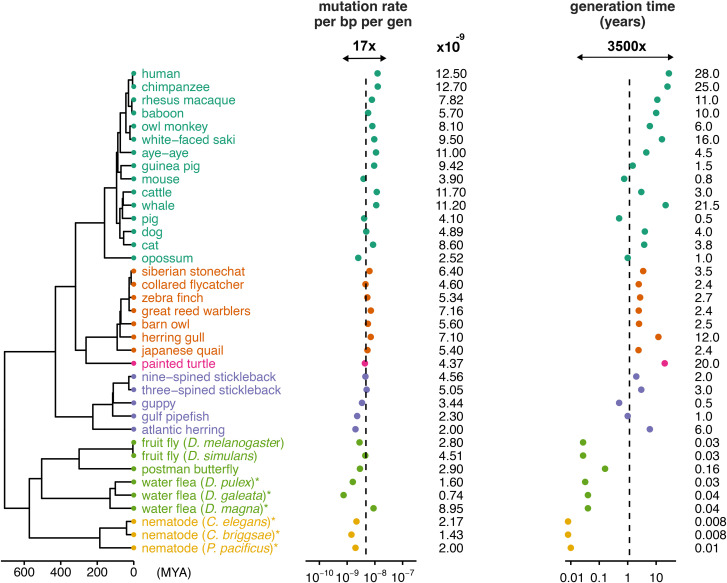
Variation in germline mutation rates per generation across animals. All estimates are based on pedigree sequencing, with the exception of mutation-accumulation experiments in worms and water fleas (marked with asterisks). Included are estimates based on a substantial fraction of the genome (in practice, >¼) and at least five trios (for pedigree-based studies). Generation time estimates are taken either from the original studies or, when not provided, from the AnAge database of animal life history traits [22]. For species with multiple independent estimates, we report the average mutation rate weighted by the number of trios in each study. The phylogeny is taken from the TimeTree database [23]; *Daphnia galeata*, which is absent from TimeTree, was added manually, with an arbitrary branch length. The data underlying this Figure, along with the data sources, can be found in S1 Table.

We collated estimates of germline mutation rates per generation obtained from whole genome resequencing of pedigrees, requiring a minimum of five trios for the estimate to be relatively precise (Box 2). Although our focus is on mammals, we included additional comparative data from animals to help place the patterns in a larger context. The picture that emerges is remarkable: despite the huge diversity of environments, metabolic rates, gametogenic processes, and three orders of magnitude variation in generation times, germline mutation rates per generation per bp all fall within roughly one order of magnitude (~10^−9^ to ~10^−8^) (Fig 1). In fact, the same order of magnitude mutation rate per generation per bp is seen even in distantly related multi-cellular organisms, such as mushrooms and plants [24].

**Box 2. Estimates of mutation rates.**

Germline mutation rates are typically estimated by resequencing the genomes of parents and their offspring, usually from blood-derived DNA. After applying filters based on allelic balance, read support, and other quality measures, researchers identify alleles present in roughly half the reads from the offspring but absent from parental genomes [21,25,26]. Standard trio sequencing captures constitutive germline mutations and some early embryonic mutations in the child, but tends to filter out early embryonic or gonosomal mutations in the parent [27]. Nevertheless, comparisons with single-cell and single-molecule sequencing in humans suggest that trio-based estimates are reliable [28–30].

Several complementary approaches have emerged that enable mutations to be assigned to different stages of the germline. Designs include sequencing of DNA from sperm, which directly samples the paternal germline [31–33], and sequencing blood-derived DNA of twins, siblings, or three-generation pedigrees, which improves sensitivity to early embryonic mutations [34–36].

A number of factors should lead estimates of the per generation mutation rate to be noisier than they are in reality. De novo mutations are rare events, so studies based on a small number of trios inevitably have large sampling errors. Variation in parental ages, especially paternal age, strongly affects the number of transmitted mutations, adding to the heterogeneity across datasets. In addition, studies differ in their filtering criteria and quality-control thresholds, which can introduce further variability [37]. At the same time, filtering pipelines are often somewhat ad hoc and may be tuned, intentionally or not, to produce mutation rates that look reasonable. Such choices can make estimates appear more similar across studies or species than they actually are. Nonetheless, the overall consistency is unlikely to be an artifact, given that phylogenetic substitution-based analyses show a similar pattern [17,38,39].

In turn, somatic mutation rates can be estimated by sequencing single cells (recently reviewed in [40]), in vitro clones [41], or, in specific settings, bulk sequencing of primary tissues. For example, Cagan and colleagues [42] sequenced individual clonal crypts, which should represent clonal outgrowths of single stem cells. Because this approach relies on bulk sequencing of the clone, it primarily captures mutations that arose in the founding stem or early progenitor cells rather than in fully differentiated cells.

**Fig 2:**
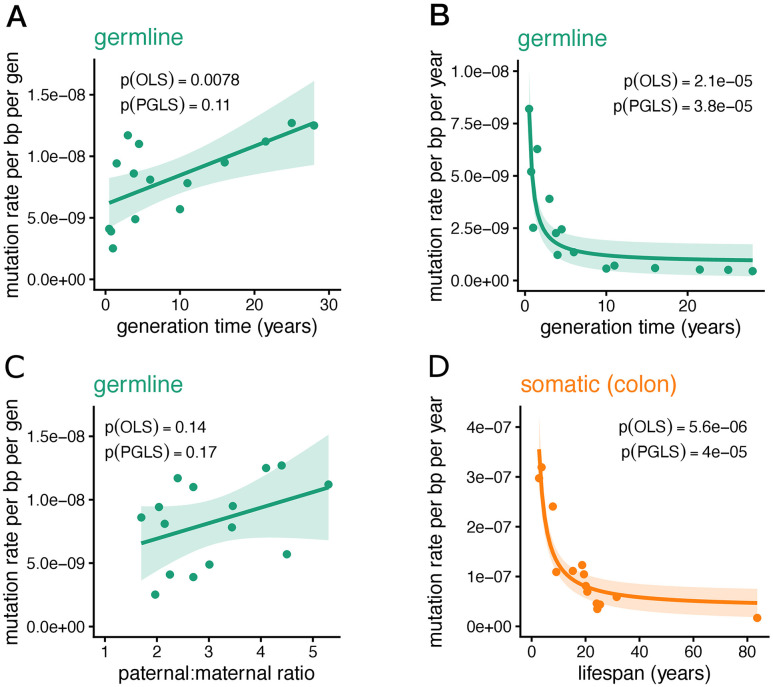
Relationships of mutation rates to generation times and paternal bias in mutation in mammals. Based on 15 mammalian species, using the same data as in Fig 1 (data can be found in S1 Table). Reported are p-values for ordinary least squares (OLS) and phylogenetic generalized least squares (PGLS) regressions [43]. Trend lines are from the OLS regression. **A**. The germline mutation rate per bp per generation shows little or no increase with generation time. **B**. The germline mutation rate per bp per year scales inversely with the generation time. **C**. No relationship is detectable between the paternal bias in mutation and the germline mutation rate per bp per generation. **D**. The mutation rate per bp per year in colonic crypts scales inversely with the life span. Intrinsic life span estimates are based on data from [42].

**Fig 3:**
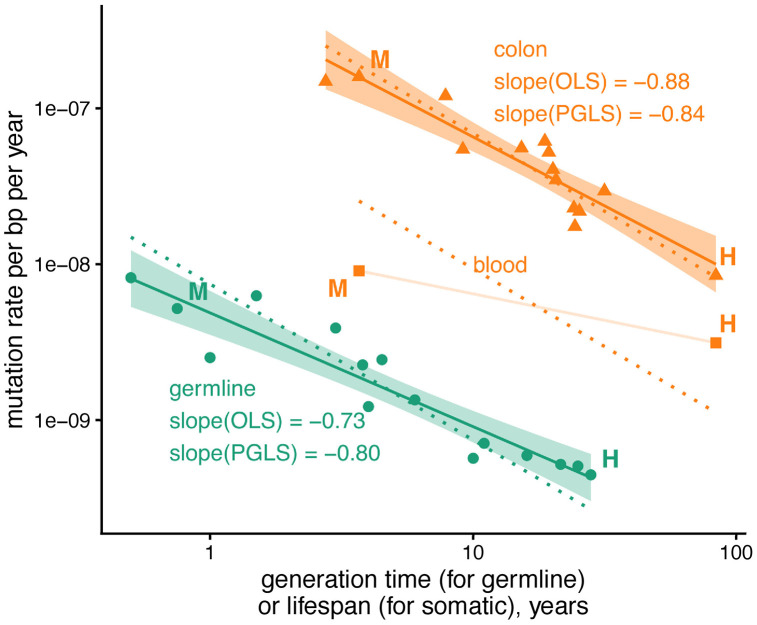
Relationship of the mutation rate per year to the generation time or life span on a log-log scale. In orange triangles are data from colonic crypts [42] and in orange squares those for blood stem cells [44,45] (human, H; mouse, M), while in blue dots are germline mutation rates (for males and females combined, as in Fig 1; data available in S1 Table). Solid lines show the fitted regression slopes obtained from ordinary least squares and shaded areas show 95% confidence intervals; dotted lines indicate a slope of −1, which corresponds to mutation rates scaling perfectly with the inverse of generation time or life span. The fainter line joining the two points serves only to illustrate that their values are not what would be expected for a slope of −1.

Given that the germline mutation rates per generation are close to constant, it follows that the rate per year is inversely proportional to generation time; we illustrate the pattern in mammals (Figs 2A, 2B, and 3), but it holds for other taxa (including invertebrates) as well [39,46]. The observation that long-lived species have a lower mutation rate per unit time is the well-known ‘generation time effect’ (Box 1) from evolutionary biology, which has been the object of study since the 1970s [15–17,47–50].

The relative constancy across species in the per generation mutation rate is all the more striking when one considers that it aggregates contributions from both sexes, different developmental stages, and distinct mutagenic processes, which differ in their proportions across species [42,51,52]. Notably, germline mutations arise early in embryogenesis, in a cell lineage that gives rise to the germline, and during gametogenesis. The relative contributions of these stages differ substantially between short- and long-lived species [20], yet the total mutation rate per bp per generation does not. Similarly, germline mutation rates are an average of maternal and paternal rates. Although the ratio of paternal to maternal germline mutations (i.e., the paternal bias; Box 1) varies slightly among species, within mammals, there is little or no correlation with the total rate (Fig 2C).

Compared to somatic cell types, the human germline mutation rate is unusually low, even relative to post-mitotic or quiescent cells: for instance, the male germline accumulates point mutations roughly 10 times more slowly than neurons [53]. But mutation rates also vary among somatic cell types: for instance, there are an additional 9 mutations per cell per year in bile ducts versus 56 mutations in colonic crypts [29].

In the one somatic tissue in which there is comparative in vivo mutation data for mammals, colonic epithelium, the same scaling is seen across species as for the germline [42]: the mutation rate per year decreases with intrinsic life span (which is highly correlated with generation time; [54]) (Figs 2D and 3). The same scaling does not appear to hold for blood stem cells, however (Fig 3). Comparing humans and mice, it does not seem to fit fibroblasts or neurons either, although there are limited data [41,55].

In summary, the mutation rate per generation seems to fall between 10^−8^ and 10^−9^ per bp across animals, despite changes in all its constituent components (Fig 1; Box 2). A small range of mutation rates is also seen across mammals in colonic crypts at the end of their life span (Fig 3) [42]. Such constancy is not expected from genetic drift alone, given the vast phylogenetic distances represented, nor is it likely to arise as a simple byproduct of life history or developmental constraints, since these differ greatly among species. Instead, the similarity points to strong selection pressures on the mutation rate per bp per generation (or life span).

As expected, if the net effect of a mutation is deleterious, there appears to be directional selection to decrease the mutation rate as much as possible. The value in a given species may be set by limits to the efficacy of selection (Box 3); alternatively, as envisioned for example by the cost of fidelity hypothesis [1,56], driving the rate down even further may inflict other costs, such that the optimum is under (indirect) stabilizing selection [57].

**Box 3. The drift barrier hypothesis and a recent extension.**

Since the average effect of a germline mutation is deleterious, on balance, an individual would be better off with no new mutations [1]. Why then do germline mutations even occur? Sturtevant, who first posed this question, speculated that perhaps it is simply not possible for an organism to prevent all mutations [11].

As Lynch [58] pointed out, however, the answer may instead lie in population genetic processes. In a small, sexually reproducing population with lots of genetic drift, a mutator allele that leads to a small number of additional mutations per generation may not inflict enough of a fitness cost on the organism for that modifier to be effectively selected against; in other words, natural selection may not overcome the ‘drift barrier’. As the effective population size (often denoted *N*_e_) increases, however, so will the efficacy of selection against the modifier allele. Thus, all else being equal, at equilibrium, species with larger *N*_e_ will have lower germline mutation rates per generation. Consistent with this expectation, per bp per generation mutation rates vary by two to three orders of magnitude between single-cell eukaryotes with huge *N*_e_ and vertebrates, in which *N*_e_ is much smaller [2,59]. Whether this theory explains variation within mammals or even vertebrates is less clear, both empirically [60] and on first principles, as there is much less variation in *N*_e_ among species than over larger phylogenetic distances.

Moreover, as well appreciated, species with different *N*_e_ also vary in a number of other potentially relevant factors. Notably, animal species with smaller *N*_e_ tend to live longer [49]. Imagine a modifier that introduces mutations, not per generation, but per year (a ‘clock-like’ modifier) and that the fitness cost is fixed across species. Then, as Zhu and colleagues [61] point out, even though there is more drift in long-lived species with small *N*_e_, the fitness cost of the modifier will be larger, because it will cause more mutations by reproduction. The net effect of these two countervailing effects—greater drift and greater selection—depends on the precise relationship between effective population sizes and generation times. But they could plausibly lead mutation rates per generation to be relatively constant or to increase only slightly with *N*_e_ values [61].

In this modeling framework, the fitness effect of a modifier allele depends on the cumulative cost of the excess deleterious mutations that it generates across the genome. Therefore, a relative constancy of mutation rates per bp across species further requires that the number of functionally important base pairs in the genome be comparable. Although this assumption is unlikely to hold exactly, it may be approximately the case: despite vast differences in the genome sizes across animals, there is roughly 2-fold variation in the number of exonic base pairs, excluding species with recent whole genome duplications (Ensembl release 115 [62]).

This modeling framework further assumes that the modifier has as its only phenotypic consequence the introduction of additional mutations. In reality, it could occasionally have beneficial pleiotropic consequences that outweigh its mutagenic effects; for example, the modifier could lead to a new cellular program that is advantageous but increases DNA damage rates. More generally, as formulated to date, the drift barrier hypothesis focuses primarily on the germline and the fitness effects of mutations transmitted to the offspring of carriers (but see [58]). As the authors of these theories note, however, modifiers of germline mutation rates are likely to also affect somatic mutation rates, imperiling the viability of their carriers [59,63]. To integrate selection pressures on both germline and soma, we need to better understand the extent to which mutation rates are coupled across cell types.

Regardless, the relatively invariant mutation rate per generation/life span (Fig 1) and the slower mutation rate per unit time in long-lived mammals (Fig 2B and 2D) are two facets of the same picture. What is not clear is whether the picture emerges from selection on the rate per generation, per year, or both.

## Mutation rates in mammals: some theories

### The view from the germline

In evolutionary biology, models for the evolution of germline mutation tend to consider selection on modifier alleles (Box 1) that increase the number of mutations inherited by offspring every generation and have no other effects [1,58]. Because, on average, a germline mutation is deleterious, such modifiers are selected against. Consistent with this expectation, the few mutator alleles mapped to date in mammals are found at a very low frequency in the population [7,64–67] and the heritability of human mutation rates is inferred to lay among rare variants [7]. The efficacy with which selection weeds mutator alleles out depends on their fitness costs and on the extent of genetic drift in the species (Box 3). Assuming all else is equal, at equilibrium, sexually reproducing species with higher levels of genetic drift are predicted to have a higher mutation rate per generation [2].

However, all is not equal: notably, species with high levels of genetic drift also tend to have longer generation times [49]. If modifier alleles introduce mutations per year (rather than per generation), then they should inflict larger fitness costs in such long-lived species, compared to shorter-lived species with more effective selection. Taking this feature into account, modeling suggests that mutation rates per generation may increase only slightly with longer generation times (and greater genetic drift; [61]). This prediction seems to fit the observations within animals (Figs 1 and 2A). However, the fact that it fits mutation rates per bp, rather than per genome, requires further explanation (Box 3).

If strong selection maintains a relatively constant per generation mutation rate, it follows that the rates per year will be inversely proportional to the generation time (Fig 2B), with no need to invoke additional selection pressures on rates per unit time. Observations in colonic crypts (Fig 2D) could then be explained either through pleiotropic effects of the repair and damage tolerance machinery active in the germline or because similar selection pressures shape (some types of) somatic cells.

### The view from the soma

Somatic mutations are the primary cause of cancers and are thought to contribute to aging by a variety of pathogenic mechanisms [4,68–74]. Notably, mutations can increase rates of cell proliferation, leading to clonal expansions that cause signaling problems, compromise tissue function, or lead to cancer [75–78]. Long-standing multistage models of carcinogenesis [79,80] posit that cancers develop once a stem cell has experienced K driver mutations (Box 1), where K depends on the tissue or cell type, reflecting differences in the physiological environment and the impact of tissue architecture on cancer initiation and promotion. Even in cases where driver mutations are not sufficient causes of cancer [81] (or even necessary causes [82]), they greatly increase the risk.

In turn, somatic mutations have long been hypothesized to drive aging [69]. A recent theory [77,83] proposes that the Gompertz law of mortality (Box 1) results from saturation in the removal of damage with age. In this framework, mutations in stem cells are considered a source of damage that gives rise to impaired differentiated cells, ultimately resulting in the accumulation of senescent cells and compromising tissue function.

If selection acts to limit carcinogenesis and the rate of aging, we would expect lower somatic mutation rates per year in longer-lived species. Accordingly, associations have been reported between the efficiency of several DNA repair pathways and life history traits in mammals [84–86] and the yearly somatic mutation rate in colonic crypts decreases with intrinsic life span [42] (Fig 2D). Depending on coevolved life strategies among species, selection pressures may depend on intrinsic life span or on life span in the wild [77]. In species with high extrinsic mortality, the somatic mutation rate will only be under selection to the limited extent that it leads to early deaths [42]. By contrast, in long-lived species with late reproduction and low fecundity or which inhabit an environmentally protected niche [77,87], selection on mutation rates may contribute to shaping life span.

Existing observations about somatic mutations across mammals are consistent with the notion that selection acts on the fitness effects of somatic mutations. If so, the analogous observations for germline mutations (Fig 2B) could be explained by pleiotropic effects on mechanisms of genome maintenance. However, if selection stems primarily from the effects of somatic mutations, it remains unclear why germline mutation rates are so much lower than those observed in somatic tissues.

### A possible synthesis

In reality, mutation rates are likely under some degree of selection in both somatic and germline cell types: for instance, the risk of cancer is greatly increased by some modifiers of germline mutation rates [7,88]. Moreover, not all somatic cell types are created equal: the effect of a mutation on organismal fitness presumably depends on the cell type function and its susceptibility to malignant transformation. Notably, one might distinguish between four cell archetypes in the body: long-lived stem cells; transient stem cells; differentiated cells that are rapidly and regularly replaced; and fully differentiated cells that do not get replaced. Long-lived stem cells can give rise to cancer or compromise tissue function via mutations (among other sources of damage). In these cells, there should be selection pressure to decrease the mutation rate per unit time, to the extent allowed by the drift barrier. In transient stem cells present only in development (e.g., neural progenitor cells, embryonic stem cells), selection pressures are likely strong, given that a large number of long-lived, descendant cells will carry any mutation that arises in them. By contrast, in differentiated cells that are rapidly and regularly replaced (e.g., neutrophils, skin fibroblasts), there is likely little direct selection pressures on mutation rates. And fully differentiated cells that do not get replaced in adulthood (e.g., neurons, cardiomyocytes, beta cells) must persist over much of the life span of an individual; in these types, as in long-lived stem cells, selection is presumably acting on the mutation rate per unit time.

From this perspective, cell types leading to gametes belong to multiple categories but are a special case in which selection is especially strong, because the mutations they carry become constitutive in the offspring. Somatic cells, meanwhile, occupy different positions along this continuum, depending on their rate of replacement and contribution to organismal fitness. Therefore, we would expect mutation rate to be lowest in germline and highest in short-lived, differentiated cells, with somatic stem cells lying somewhere in between.

In this respect, it is intriguing that the patterns observed across mammals (i.e., the slopes in Fig 3) point to analogous selection pressures on the germline and colonic crypts across species. Specifically, the observed relationships between mutation rates per unit time and generation time (or life span) suggest that the fitness cost of mutators is proportional to generation time (or life span) and similar across mammals, as might be the case if most modifiers introduce mutations per unit time [61]. However, a similar scaling does not seem to hold for all cell types. In mouse blood stem cells, the yearly mutation rate is lower than expected relative to humans (or equivalently, there is an unexpectedly high rate in humans; Fig 3). This observation may be explained by different selection pressures on mutations in the two species, arising from distinct effects of mutations on cancer risk (e.g., because blood cancer is initiated by fewer driver mutations or more readily in mice than in humans [89]), a larger numbers of sites in mice at which mutations compromise function, or stronger pleiotropic effects of mutation modifiers (either within the cell or on other cell types) in the mouse.

## Possible mechanistic underpinnings

Recent evidence suggests that most point mutations in the germline and soma can be explained by two ubiquitous COSMIC mutational signatures (Box 1): single base substitution (SBS) signature SBS1 (which consists of transitions at methylated CpGs due to deamination and/or replication errors) and an SBS5-like flat signature (potentially caused by collateral errors during translesion synthesis and repair) [29,52,90,91]. Both of these signatures are generally ‘clock-like’ in that they accumulate linearly with age [30,92]. Other mutational processes are not well captured by COSMIC signatures, such as the specific C > G substitutions enriched in aging oocytes in apes [93]. In several somatic cell types, there is also evidence of other known damage-induced processes, such as mutations due to oxidative damage or UV radiation [29,42]. Overall, in the vertebrate soma and germline, mutations in long-lived cells (such as stem cells or neurons) seem to be predominantly caused by DNA damage and increase linearly over time [30,75,92]. We note, however, that a fraction of mutations carried by a cell originate during short-lived cell states, such as embryogenesis, or in rapidly replaced differentiated types.

In principle, for selection to reduce the mutation rate in a cell type, it could act to increase repair accuracy (e.g., the error rate of a polymerase [94]), increase repair efficiency (e.g., the abundance of repair enzymes [95]), or decrease DNA damage rates (e.g., by increasing melanin production in skin). Because a number of lesion types lead to mutations owing to error-prone genome replication, slowing down cell division may also increase repair efficiency, all else being equal [96]. In addition to preventing the accrual of mutations, selection can target mechanisms to reduce the consequences of mutations (e.g., by promoting the removal of mutated cells [97]), by evolving multi-layered anti-tumorigenic mechanisms [98,99], or by developing tissue architectures that reduce the chance of uncontrolled clonal expansions [100].

Some strategies for reducing the mutation rate will be shared among cell types, while others may be specific to a cell type or tissue. If a few key genes influence the repair accuracy, changes to such genes will likely be highly pleiotropic, influencing mutation rates across many cell types. This broad impact increases the fitness cost of modifiers that decrease accuracy, making them more likely to overcome the drift barrier (Box 3). By contrast, modifiers of DNA damage rates may be restricted in their effects to specific cell types or tissues, given that DNA damage likely depends on cellular programs and metabolism, as well as on exposures to mutagens that are environment specific. Thus, depending on the role of the cell, there may be varying levels of selection to prevent the effects of DNA damage, and in some species, greater functional costs to doing so (e.g., increasing melanin could decrease vitamin D synthesis [101]). These considerations suggest that differences in repair accuracy across species may be primarily driven by the efficacy of selection and at the drift barrier (Box 3), whereas the extent of DNA damage in any given cell type, and the selection pressures to reduce it, may be more variable among species.

## Conclusions and future directions

To date, theories about selection pressures on mutation have been developed largely in parallel in different fields, with evolutionary biologists focused on the germline and emphasizing the role of population processes of selection and drift, and cancer biologists and aging specialists highlighting the deleterious consequences of mutations in the soma. Moreover, while existing models often rely on assumptions that mutations arise from replication errors or track cell divisions [58,77,102], recent evidence suggests that most mutations in the germline and soma are triggered by endogenous and exogenous sources of damage and can arise independently of cell divisions [6,30,53,91,103]. There is therefore a need for a new theory that integrates these recent findings and knowledge of tissue architecture into models for selection on mutation rates and contends with the pleiotropic effects that couple mutagenic processes across cell types.

In this regard, it may be helpful to think of selection on mutation rates not only in terms of the dichotomy of germline versus soma, but as a continuum of fitness effects, with constitutive mutations at one end and mutations in fully differentiated, short-lived cell types likely at the other. Ultimately, predicting the mutation rate of a cell type in a given species will likely require a framework that includes the developmental trajectory of the cell, its longevity, and other properties that influence its contribution to organismal fitness. For a specific cell type, the selection pressures on modifier alleles will depend on features like the mutational target size that can drive cancer or lead to incorrect sensing of hormone levels, as well as on pleiotropic consequences.

As suggested by evolutionary models, the scaling pattern observed in the germline and colonic crypts of mammals, in which mutation rates per year are inversely proportional to generation time or life span (Fig 3), is expected to arise when mutations inflict a similar fitness cost per year across species. However, not all cell types are expected to meet these conditions: in some, mutations are likely inconsequential to organismal fitness and mutation rates will only be constrained by pleiotropic effects in other cell types while, in others, the fitness consequences of the same yearly mutational burden will differ among species, or the main influence will come from modifier alleles that introduce mutations per generation rather than per unit time. Consequently, we predict that as somatic mutation data becomes available for a wider range of species and tissues, deviations from this scaling will emerge. Conversely, we should learn a great deal about selection pressures shaping the different cell types from such comparative data.

## Supporting information

S1 Table Germline mutation rate estimates across species. For each species included in Fig 1, we report the per generation mutation rate, number of trios (for pedigree-based studies) or lines (for mutation-accumulation studies), generation time, source publication, and, if available, the degree of paternal bias.
(XLSX)

## Abbreviations:

COSMIC: catalog of somatic mutations in cancer
OLS: ordinary least squares
PGLS: phylogenetic generalized least squares
SBS: single base substitution
